# Data-driven nucleus subclassification on colon hematoxylin and eosin using style-transferred digital pathology

**DOI:** 10.1117/1.JMI.11.6.067501

**Published:** 2024-11-05

**Authors:** Lucas W. Remedios, Shunxing Bao, Samuel W. Remedios, Ho Hin Lee, Leon Y. Cai, Thomas Li, Ruining Deng, Nancy R. Newlin, Adam M. Saunders, Can Cui, Jia Li, Qi Liu, Ken S. Lau, Joseph T. Roland, Mary K. Washington, Lori A. Coburn, Keith T. Wilson, Yuankai Huo, Bennett A. Landman

**Affiliations:** a Vanderbilt University, Department of Computer Science, Nashville, Tennessee, United States; b Vanderbilt University, Department of Electrical and Computer Engineering, Nashville, Tennessee, United States; c Johns Hopkins University, Department of Computer Science, Baltimore, Maryland, United States; d National Institutes of Health, Department of Radiology and Imaging Sciences, Bethesda, Maryland, United States; e Vanderbilt University, Department of Biomedical Engineering, Nashville, Tennessee, United States; f Vanderbilt University Medical Center, Department of Biostatistics, Nashville, Tennessee, United States; g Vanderbilt University Medical Center, Center for Quantitative Sciences, Nashville, Tennessee, United States; h Vanderbilt University Medical Center, Epithelial Biology Center, Nashville, Tennessee, United States; i Vanderbilt University School of Medicine, Department of Cell and Developmental Biology, Nashville, Tennessee, United States; j Vanderbilt University Medical Center, Department of Pathology, Microbiology, and Immunology, Nashville, Tennessee, United States; k Vanderbilt University Medical Center, Division of Gastroenterology, Hepatology, and Nutrition, Department of Medicine, Nashville, Tennessee, United States; l Vanderbilt University Medical Center, Vanderbilt Center for Mucosal Inflammation and Cancer, Nashville, Tennessee, United States; m Vanderbilt University School of Medicine, Program in Cancer Biology, Nashville, Tennessee, United States; n Veterans Affairs Tennessee Valley Healthcare System, Nashville, Tennessee, United States

**Keywords:** hematoxylin and eosin, multiplexed immunofluorescence, cell classification, virtual hematoxylin and eosin, domain shift, virtual staining

## Abstract

**Purpose:**

Cells are building blocks for human physiology; consequently, understanding the way cells communicate, co-locate, and interrelate is essential to furthering our understanding of how the body functions in both health and disease. Hematoxylin and eosin (H&E) is the standard stain used in histological analysis of tissues in both clinical and research settings. Although H&E is ubiquitous and reveals tissue microanatomy, the classification and mapping of cell subtypes often require the use of specialized stains. The recent CoNIC Challenge focused on artificial intelligence classification of six types of cells on colon H&E but was unable to classify epithelial subtypes (progenitor, enteroendocrine, goblet), lymphocyte subtypes (B, helper T, cytotoxic T), and connective subtypes (fibroblasts). We propose to use inter-modality learning to label previously un-labelable cell types on H&E.

**Approach:**

We took advantage of the cell classification information inherent in multiplexed immunofluorescence (MxIF) histology to create cell-level annotations for 14 subclasses. Then, we performed style transfer on the MxIF to synthesize realistic virtual H&E. We assessed the efficacy of a supervised learning scheme using the virtual H&E and 14 subclass labels. We evaluated our model on virtual H&E and real H&E.

**Results:**

On virtual H&E, we were able to classify helper T cells and epithelial progenitors with positive predictive values of 0.34±0.15 (prevalence 0.03±0.01) and 0.47±0.1 (prevalence 0.07±0.02), respectively, when using ground truth centroid information. On real H&E, we needed to compute bounded metrics instead of direct metrics because our fine-grained virtual H&E predicted classes had to be matched to the closest available parent classes in the coarser labels from the real H&E dataset. For the real H&E, we could classify bounded metrics for the helper T cells and epithelial progenitors with upper bound positive predictive values of 0.43±0.03 (parent class prevalence 0.21) and 0.94±0.02 (parent class prevalence 0.49) when using ground truth centroid information.

**Conclusions:**

This is the first work to provide cell type classification for helper T and epithelial progenitor nuclei on H&E.

## 1 Introduction

Hematoxylin and eosin (H&E) stains are ubiquitous in pathology.^1^ The H&E staining causes cell nuclei to turn blue and other tissue to turn pink.^2^ Unfortunately, accurately identifying fine details of microanatomy on H&E-stained samples is challenging for those without pathology expertise,^3^ which makes large-scale manual annotation of subtle structures costly and time-intensive.

To counter the limitations of manual annotation on H&E, deep learning has been proposed as an alternate and automatic method for labeling microanatomy.^4^ Deep learning algorithms are data-hungry, meaning that performance generally improves as datasets increase in size.^5^ Because manual annotation of cells is expensive and slow, the public release of large, labeled datasets in this space is important to facilitate the training of automatic cell identification algorithms. In 2022, the CoNIC Challenge released a dataset of colon H&E with six nucleus cell type annotations.^3^^,^^6^ The challenge data were annotated using a complicated and repetitive approach that involved both automatic nucleus annotation and refinement based on feedback from trained pathologists.^3^^,^^6^ Cell segmentation is an important and popular topic in digital pathology, with segmentations being useful in downstream applications.^4^^,^^7^^–^^12^ The development of automatic and reliable nucleus classification algorithms for H&E slides would allow for more comprehensive large-scale cell mapping, which promises a better understanding of human physiology in both health and disease.

In contrast to H&E, multiplexed immunofluorescence (MxIF) imaging directly enables subclassification of cells. MxIF involves the staining and imaging of the same tissue multiple times via bleaching and re-staining.^13^ When many stains are used in MxIF, a more detailed understanding of tissue structure can be attained than what is available in H&E because different stains can bind to different subsets of the tissue. When many stains are used on the same tissue, nuclei/cells can be classified based on which combinations of stains bind to each nucleus/cell.

The digital synthesis of unacquired stains is known as virtual staining.^14^ Image slides can be virtually stained when the starting point is label-free or an acquired stain.^15^ Taking an image of stained tissue and computationally generating a virtually stained image of the same tissue is known as stain-to-stain transformation.^15^ Previous studies have used deep learning generative adversarial networks (GANs)^16^ and conditional GANs^17^ to synthesize virtual H&E images.^18^^–^^20^ In this work, we refer to both synthetic and real data. To distinguish between acquired H&E and synthesized H&E, we henceforth refer to these as real H&E and virtual H&E, respectively.

MxIF is a specialized technology, making these images rare, whereas H&E is ubiquitous. It would be beneficial to derive the intricate microanatomical details present in MxIF images from H&E samples. Bridging the gap between MxIF and H&E can be formulated as a computational problem.

Co-leveraging H&E and MxIF information has been performed in several studies. Nadarajan et al.^21^ performed semantic segmentation of simple structures on real H&E using MxIF-derived labels from the same tissue with paired H&E and MxIF stains. In a follow-up paper, the same group used a conditional GAN to create virtual H&E from MxIF.^2^ A semantic segmentation model was then trained on the virtual H&E with MxIF-derived labels to semantically segment four simple structures (i.e., all nuclei, cytoplasm, membranes, background) and evaluated on real H&E. Further work has been conducted in this area with Han et al.^22^ having designed a model that learned to classify four types of cells (i.e., ER+, PR+, HER2+, and Ki67+) from real H&E by leveraging paired MxIF information.

**Fig. 1:**
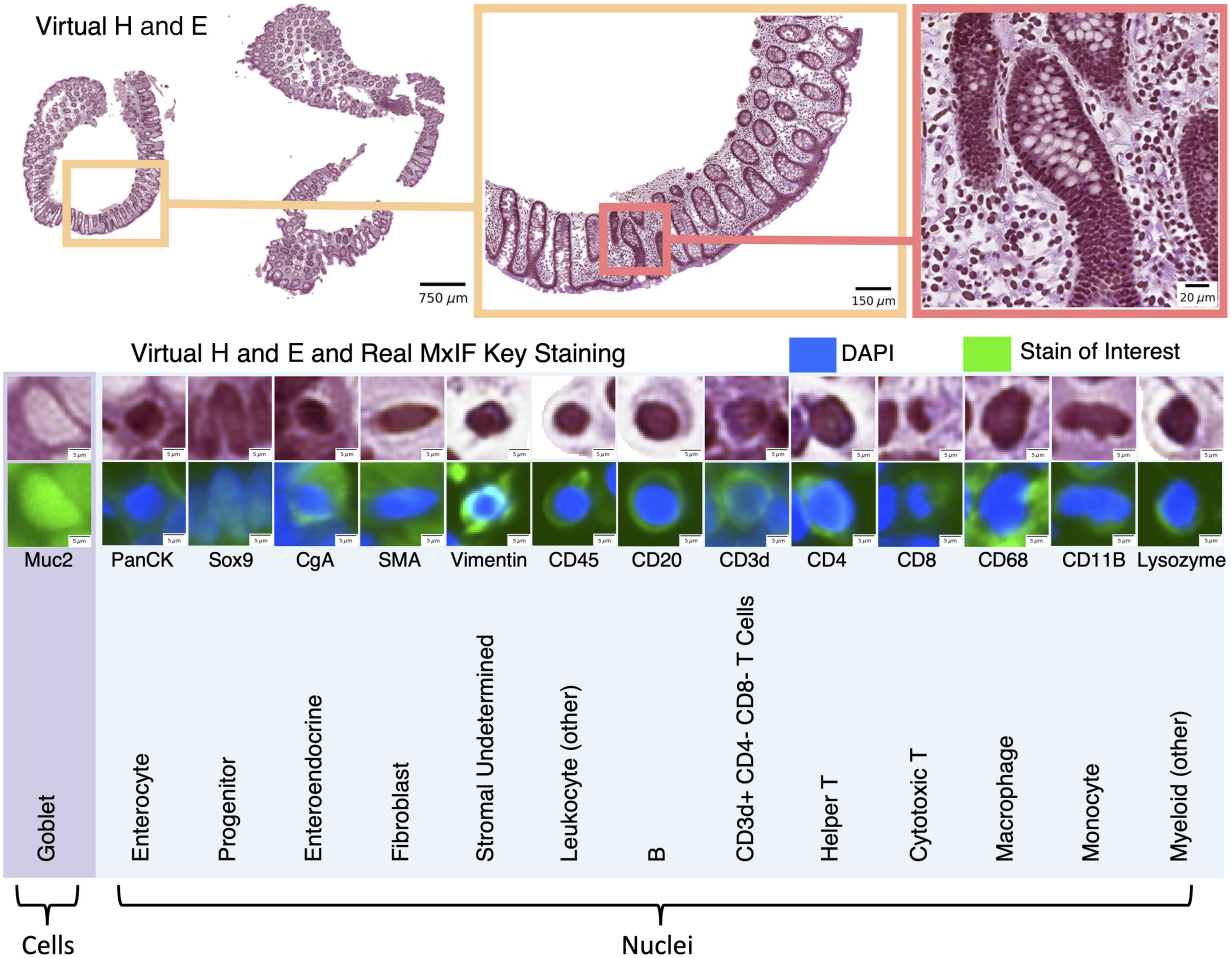
We leveraged inter-modality learning to investigate the identification of nuclei and cells on H&E staining that are traditionally viewed with specialized staining. The realistic quality of our virtual H&E holds at multiple scales (top section). Representative nuclei and cells from each of our 14 classes in both virtual H&E and MxIF illustrate intensity and morphological variation across cell types (lower section). Green is used to denote the MxIF stain of interest, which is a different stain for each of the 14 classes in this figure. Although the signal to identify these classes of nuclei and cells is present in MxIF, the classes are more difficult to distinguish on virtual H&E.

We investigate the more difficult task of learning to subclassify nuclei and cells into 14 categories on virtual H&E (Fig. 1) and evaluate our models on real H&E from a public dataset. Our work is a part of the Gut Cell Atlas Crohn’s Disease Consortium, which aims to map intestinal cells.^23^^,^^24^ The staining data acquired in this work for cell/nucleus subclassification were specifically chosen by the clinical members of our team to support Crohn’s disease intestinal cell mapping. The contributions of this work are: (1) we demonstrate the degree with which 14 categories of nuclei/cells can be simultaneously learned on virtual H&E when using paired cell subclassification labels in a supervised training scheme, (2) we demonstrate the degree with which the virtual-trained model generalizes to real H&E, and (3) we are the first to automatically identify helper T and epithelial progenitor nuclei on H&E. This paper is a considerable extension of our prior work.^25^

## 2 Methods

**Fig. 2:**
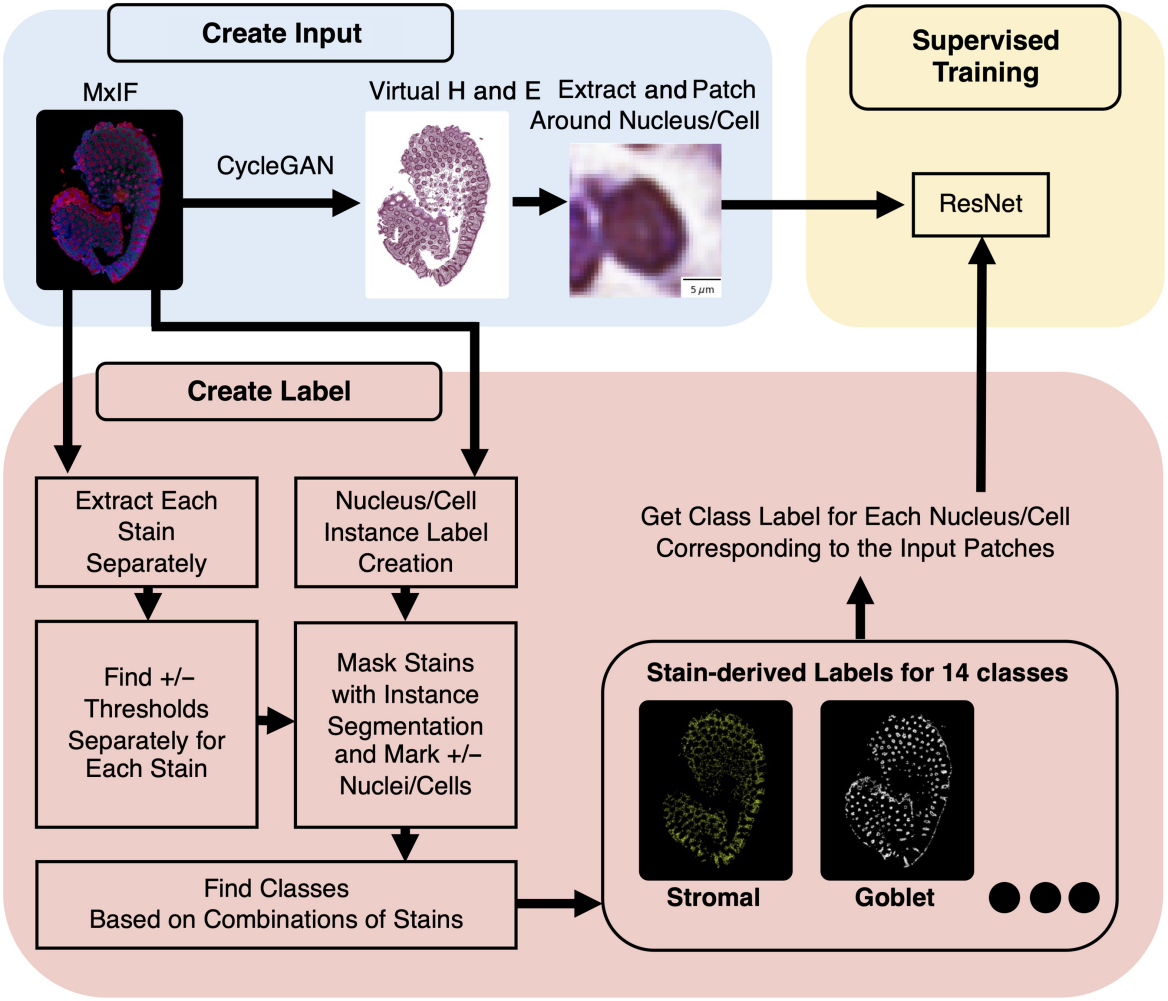
Our approach is similar to Han et al.,^22^ with the additions of learning classification from virtual data and targeting the classification of 14 nucleus/cell classes rather than 4.

The goal of this paper was to segment and classify 14 types of cells/nuclei in colon tissue on H&E. We proposed to use style transfer from MxIF to virtual H&E to permit supervised learning (Fig. 2). To train a cell classification model that could later be applied to unlabeled H&E data, we needed to have a pipeline that consisted of two major components: nucleus/cell localization and nucleus/cell classification. In this pursuit, our pipeline consisted of an instance segmentation model and a separate classification model (on image patches surrounding each nucleus/cell).

We studied an in-house dataset of MxIF images that were stained with 27 markers and used the inherent cell classification information to train a model to identify these cell categories on virtual H&E. We further evaluated the models on real H&E.

In more detail, the MxIF images were style transferred to the H&E domain (Sec. 2.4). We used deep learning in a supervised training approach to learn to classify 14 types of nuclei and cells on virtual H&E in a multi-class classification approach (Secs. 2.5–2.7). The class label information was obtained for each cell or nucleus from the MxIF by using classification rules from biological domain knowledge about how combinations of markers bind to cells.^25^ Specifically, labels were generated for nucleus/cell classes via combinations of 17 out of 27 MxIF stains (Sec. 2.3 and Table 1). The virtual H&E was synthesized by using a CycleGAN^26^ on all 27 MxIF stains.

**Table 1:** Mapping of the subset of key stains from the 27 MxIF stains that enable classification of cells/nuclei in 14 classes.

Nucleus/cell subtype	Key stain(s)
All nuclei	DAPI
Goblet	Muc2
Enterocyte	PanCK, NaKATPase
Progenitor	Sox9, OLFM4
Enteroendocrine	CgA
Fibroblast	SMA
Stromal undetermined	Vimentin
Leukocyte (other)	CD45
B	CD20
CD3d+ CD4− CD8− T	CD3d
Helper T	CD4
Cytotoxic T	CD8
Macrophage	CD68
Monocyte	CD11b
Myeloid (other)	Lysozyme

We trained a ResNet-18^27^ on 41×41  pixels (20.5×20.5  μm) image patches of virtual H&E with one nucleus or cell centered in each patch. The class label for each image patch corresponded to the center nucleus/cell and was derived from the stain combinations on the same nucleus or cell in the MxIF. We evaluated the models on both withheld virtual H&E, as well as public real H&E from a multi-site dataset.

### 2.1 In-House MxIF Data

Samples were studied in deidentified form from Vanderbilt University Medical Center under Institutional Review Board approval (IRB #191738 and #191777). The samples were labeled at the slide level by a pathologist as normal, quiescent, mild, moderate, or severe, with respect to Crohn’s disease activity. The samples were formalin-fixed and paraffin-embedded. We used 28 whole slides imaged at 0.32  μm per pixel (14 from the ascending colon and 14 from the terminal ileum) from 20 patients. Not every sample from a Crohn’s disease patient depicts the disease.

Of the 28 slides, six were control tissue, six were normal tissue, six were quiescent Crohn’s disease, six were mild Crohn’s disease, two were moderate Crohn’s disease, and two were severe Crohn’s disease. The distribution of slides per patient was as follows: 14 patients had one slide, five patients had two slides, and one patient had four slides. Slides were split at the patient level during training, validation, and testing, which we discuss in further detail in Sec. 2.7. We studied 17 out of 27 stain channels to annotate cell types on MxIF: NaKATPase, PanCK, Muc2, CgA, Vimentin, DAPI, SMA, Sox9, OLFM4, Lysozyme, CD45, CD20, CD68, CD11B, CD3d, CD8, and CD4 (Table 1). Although we used 17 stains for annotation, we annotated 14 classes because each of the 17 stains did not always directly map to a unique nucleus/cell type. These stains came from a subset of a previously described protocol.^20^

### 2.2 Public Real H&E Data

The public real H&E data came from the CoNIC Challenge 2022 and is from normal colon tissue as well as colon tissue depicting cancer, dysplasia, and inflammation.^6^ There were 4981 image patches of size 256×256  pixels, which came from images with a resolution of ∼0.5  μm per pixel. Nuclei in this dataset had labels for six cell types: epithelial, lymphocyte, plasma, eosinophil, neutrophil, and connective. The data came from multiple sites in the United States, England, and China.^3^

Moreover, the data were aggregated from five source datasets: CRAG,^28^ GlaS,^29^ DigestPath, PanNuke,^30^ and CoNSeP.^31^

### 2.3 Label Generation Leveraging MxIF

The multi-stain nature of MxIF allows cell subclassification by design, which we leveraged to automatically generate our 14 class labels. Generating classification labels for nuclei and cells required identifying whether a stain bound or did not bind to cells at the whole slide image level. To determine stain binding required picking a threshold for positive and negative nuclei and cells, for each stain. In this work, a senior digital pathology researcher manually selected and applied stain-wise thresholds. These thresholds were determined separately by stain channel for each MxIF whole slide image. Thresholds were determined on images with autofluorescence removed but without further preprocessing such as normalization.

To classify the nuclei, we needed to know their locations. The MxIF did not have cell centroids labeled, so we opted for an automatic approach. To determine the location of each nucleus/cell, we obtained an instance segmentation by performing inference with the pretrained DeepCell Mesmer model.^32^ We passed the Mesmer model a single grayscale channel image as input. This single channel input was the sum of the MxIF DAPI and Muc2 channels. Merging the channels through addition was reasonable because DAPI identifies nuclei, and Muc2 identifies the goblets associated with goblet cells. Each nucleus or cell was then categorized as positive or negative for each stain type by computing its mean stain intensity and applying the manual thresholds. We assigned each instance a single class label based on a series of biological rules.^25^ The key stains needed to identify the cell/nucleus types are highlighted in Table 1. After label generation, we had 14 classes, 13 of which were nucleus classes: enteroendocrine, enterocyte, epithelial progenitor, fibroblast, stromal (undetermined), monocyte, macrophage, helper T, cytotoxic T, CD3d+ CD4− CD8− T, B, myeloid (other), and leukocyte (other), and one of which was a cell class: goblet. Each cell/nucleus in the dataset had one single label. Although leukocyte and myeloid are parent classes for some of the other cell types, a cell with the label leukocyte or myeloid was unable to be identified with any more detail given the ground truth staining information for that nucleus. As such, we refer to leukocyte as leukocyte (other) and myeloid as myeloid (other).

We differentiate goblet cells as not being nuclei because they were identified via MUC2 positive regions, which are the goblets from goblet cells, rather than the nuclei of the goblet cells. For progenitors, as 96% were epithelial-positive in our dataset, we refer to progenitors and epithelial progenitors interchangeably throughout this paper. In addition, the nucleus class stromal (undetermined) refers to cells in the stroma that were not classified further into any specific subclasses.

### 2.4 Virtual H&E Via Style Transfer

As our goal was to segment and classify from H&E data, we used a pretrained network to style transfer the MxIF (from which the cell classification labels were derived) to virtual H&E. The virtual H&E was inferred from all of the available 27 MxIF stains for the 28 MxIF whole slide images. We used a pretrained CycleGAN-based model that performed style transfer. This model was trained on the same 28 whole slide images of in-house MxIF and 28 close-cut tissue slices of in-house real H&E. Close-cut refers to adjacent tissue slices cut from the same block of tissue. The architecture and training strategy [named “Proposed-(8)”] were described in detail in a previous work from our team.^20^ In our paper, the style transfer from MxIF to virtual H&E was performed using the pretrained “Proposed-(8)” model, exactly as described by Bao et al.^20^

### 2.5 Segmentation Model and Training Approach

The instance segmentation model we used on virtual H&E was a Hover-Net.^31^ We trained the Hover-Nets on virtual H&E input. The matching instance segmentation labels were created from another prediction model, not from a human rater or a human-in-the-loop labeling approach. The labels were created using predictions from the pretrained DeepCell Mesmer model on MxIF. The MxIF data were downsampled via cubic interpolation to 0.5  μm per pixel so that the Hover-Nets would work at the same resolution as our target real H&E data.^3^^,^^6^ The training strategy that we selected was the default from the Hover-Net public GitHub repository: https://github.com/vqdang/hover_net.

### 2.6 Classification Model and Training Approach

We selected ResNet-18^27^ as our classification model. The model was initialized from the PyTorch 1.12.1 default ResNet-18^27^ pretrained on ImageNet.^33^ To remove negative effects from class imbalance and batch size differences between training and testing, we replaced each batch normalization^34^ layer with instance normalization.^35^ The instance normalization layers were implemented with PyTorch using default parameters.

The ResNet-18 was trained for classification on image patches of virtual H&E. The patches were resampled via cubic interpolation to a standard H&E resolution of 0.5  μm per pixel. Our patch extraction strategy involved selecting patches of size 41×41  pixels centered on a nucleus or cell. All patches were individually intensity normalized between 0 and 1. A single class label was assigned to each patch, corresponding to the center nucleus or cell (Fig. 1). To address heavy class imbalance during training, at each batch, we ensured that each example had an equal likelihood of coming from any of the 14 classes. The model was trained for 20,000 steps using a batch size of 256, the Adam optimizer,^36^ a learning rate of 0.001, cross-entropy loss, and the one-cycle learning rate scheduler.^37^

All of the classification code was implemented in Python 3.8 using PyTorch 1.12.1 and Torchvision 0.13.1. In addition, all classification training and inference were performed using an Nvidia RTX A6000.

### 2.7 Cross-validation and External Validation

**Fig. 3:**
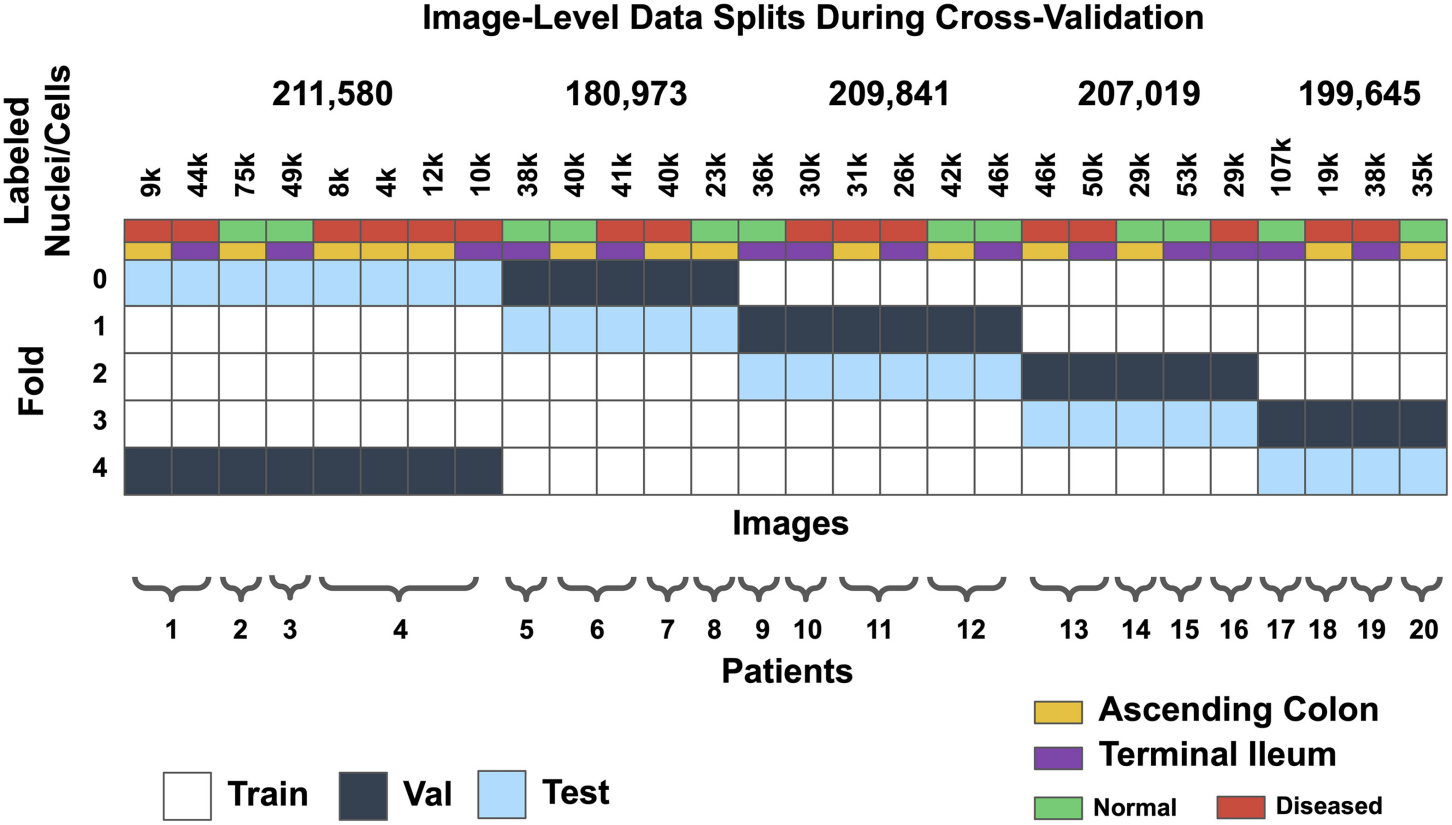
To balance the quality of the models trained on each fold of data, we split training, validation, and testing data at the patient level (each column is an image). In addition, each training, validation, and testing fold contained tissue without Crohn’s disease activity (normal) and tissue with Crohn’s disease activity (diseased) from the ascending colon and terminal ileum.

We performed five-fold cross-validation separately on both the instance segmentation and classification models on the virtual H&E. To avoid data contamination, the training, validation, and testing data were split at the patient level (Fig. 3). To maintain consistency across folds, we specified that in each fold, the training data contained 12 patients, validation contained four patients, and testing contained four patients. To reduce bias, we always included data from both the ascending colon and terminal ileum, healthy and diseased, in each training, validation, and testing set. The folds and splits used for training, validation, and testing were the same for the instance segmentation model and the classification model.

As mentioned in Sec. 2.3, on our virtual H&E we created labels for goblet cells, which we identified by finding the Muc2+ goblets associated with the cells, rather than by finding their nuclei. Goblets look very different from cell nuclei on H&E (Fig. 1). On the real H&E data that we selected for external validation, there were no labels on the goblets associated with goblet cells because these data came from the CoNIC Challenge, which only dealt with nuclei. Hence, we needed to train two instance segmentation models (Hover-Nets): the first to segment nuclei and goblets so that we could evaluate our approach on our virtual H&E, and the second to only segment nuclei so that we could evaluate the real H&E from the CoNIC Challenge.

We selected weights for evaluation based on the step with the lowest validation loss for each fold. The evaluation was performed on the segmentation and classification models both separately and combined.

Our entire cross-validation approach was performed on virtual H&E. To test how well our virtual-trained models generalized to unseen real H&E data, we used the publicly available CoNIC dataset.^6^ This dataset contained 4981 image patches from five source datasets. To reduce the effects of the domain shift between the stain color of our virtual H&E and the real H&E data, we trained separate CycleGANs to make the real H&E staining more similar to our virtual H&E.

The CycleGANs to correct H&E stain differences were trained using a sample of 20,000 patches of virtual H&E as well as the real H&E patches from the CoNIC dataset. Due to systematic staining differences from the different sites within the CoNIC dataset, a separate CycleGAN was trained for each site within the CoNIC dataset, which resulted in five CycleGANs (one for each site). The GANs were trained for 100 epochs using a batch size of 2 and a learning rate of 0.0002. All patches used in training the stain-correcting CycleGAN were of size 256×256  pixels and at a resolution of 0.5  μm per pixel. The weight selection was determined based on the qualitative evaluation of the ability of each CycleGAN to realistically change the stain color in one direction, from the specific CoNIC site stain color to the virtual H&E stain color. The style-transferred patches of real H&E were then used for external validation of the segmentation and classification approach.

## 3 Results

### 3.1 Results for Nucleus/Cell Instance Segmentation

**Fig. 4:**
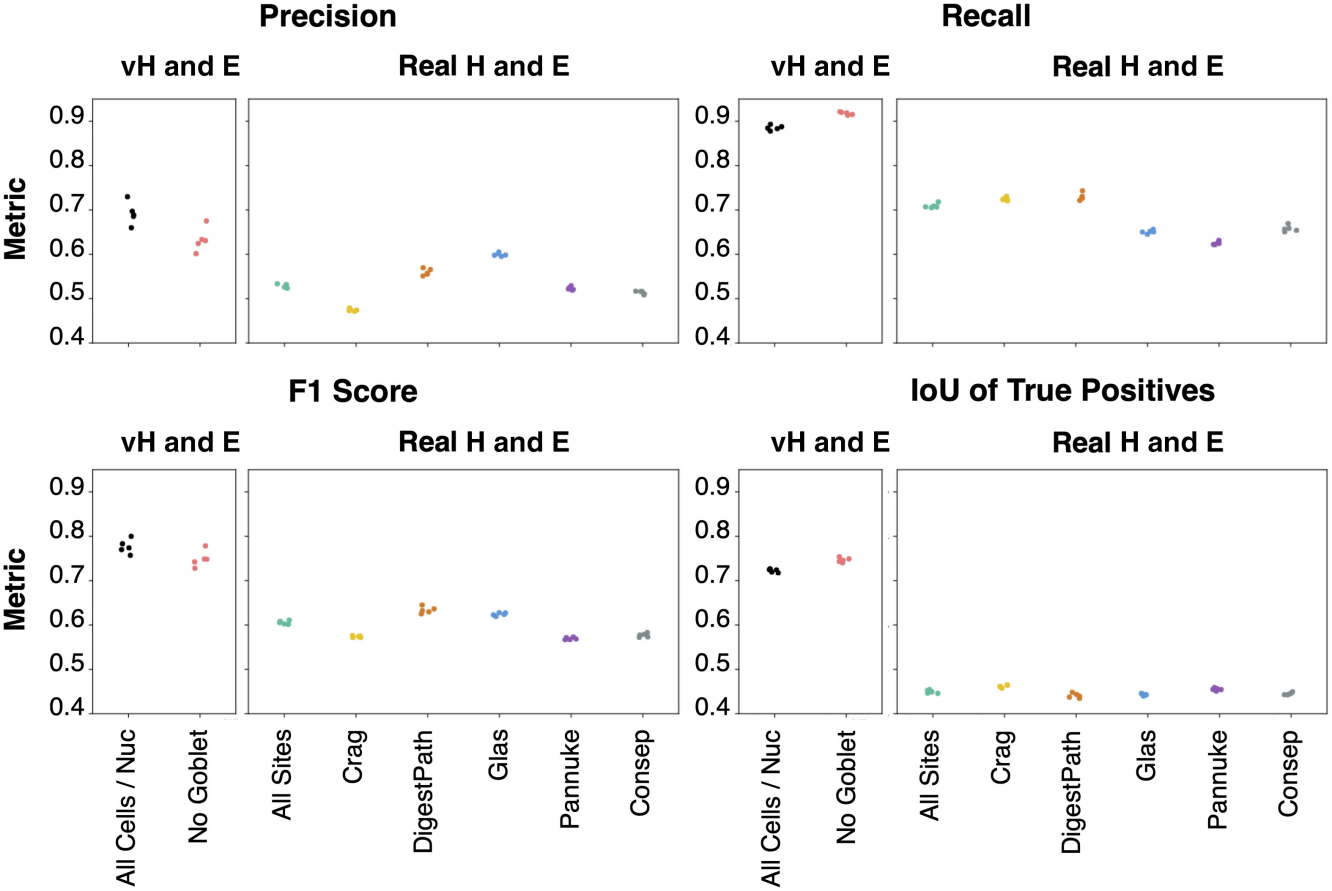
Instance segmentation was better on recall than precision due to a lower number of false negatives than false positives. A drop in performance across metrics on real H&E highlights domain shift. The performance gap for intersection over union (IoU) of true positives implies that the size and shapes of predicted nuclei/cells more closely matched the virtual H&E than the real H&E. The five data points shown for each metric are from the five folds of cross-validation—each datapoint is the mean of the metrics computed at the nucleus level for that fold.

The instance segmentation was evaluated with precision, recall, F1 score, and intersection over union (IoU) of the true positives (Fig. 4). We defined true positives as nuclei/cells with an IoU greater than 0.25 between the prediction and the label. In the true positives, each label was only matched to a single prediction, and each prediction was only matched to a single label. If there was no matching label for a prediction, then the nucleus/cell was a false positive. If a label had no matching prediction, then the nucleus/cell was a false negative. Defining true positive, false positive, and false negative using a lenient IoU threshold of 0.25 was necessary to account for the differences in the label sets between our virtual H&E and real H&E. More specifically, the instance segmentation labels on our virtual H&E (inferred by the pretrained DeepCell Mesmer model) were not as closely cropped to the boundary of each nucleus/cell as the labels on the real H&E from the CoNIC dataset.

On the virtual H&E and real H&E, recall was better than precision (Fig. 4). In addition, on virtual H&E, when goblet cells were included, precision increased compared with when goblet cells were excluded. On the real H&E, there was performance variability due to the site.

**Fig. 5:**
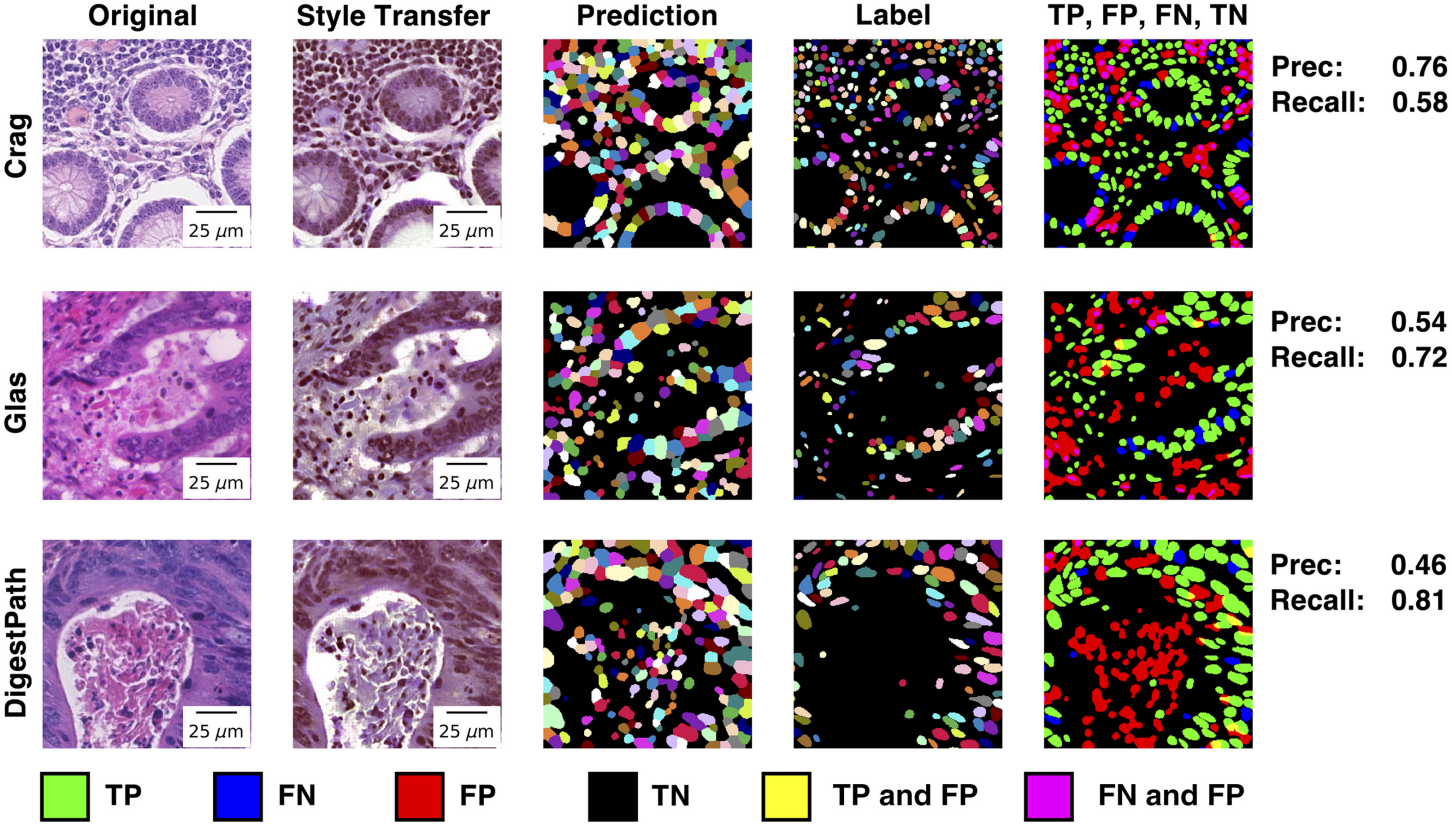
To counteract domain shift in H&E staining, instance segmentation was performed on a style-transferred version of the real H&E. In the column titled: “TP, FP, FN, TN”, nuclei were matched via a lenient IoU threshold of 0.25, as described in Sec. 3.1. True positive (green) and false negative (blue) nuclei were plotted based on the segmentations provided in the ground truth labels. Because of the lenient IoU threshold, false positive (red) nuclei (which were predictions rather than labels) could have some pixel overlap with ground truth labels. In areas where pixel overlap occurred between false positives (predictions plotted) and false negatives or true positives (labels plotted), we mixed the individual RGB colors, resulting in these additional plotted categories: “TP & FP” in yellow, and “FN & FP” in magenta. Lower precision on instance segmentation occurred when (1) the labels were not exhaustive and (2) style transfer made tissue darker and presented more like nuclei. Note that the colors for nuclei are not expected to match between the prediction and label columns—these colors were used to visually separate instances but do not have any classification meaning.

In Fig. 5, we further investigated the instance segmentation on the real H&E through visualization. The model sometimes confused darker-colored style transferred structures with nuclei. In addition, the labels on the real H&E did not always contain every nucleus. Both factors contributed to a higher number of false positives.

### 3.2 Results for Virtual H&E Nucleus/Cell Classification

**Fig. 6:**
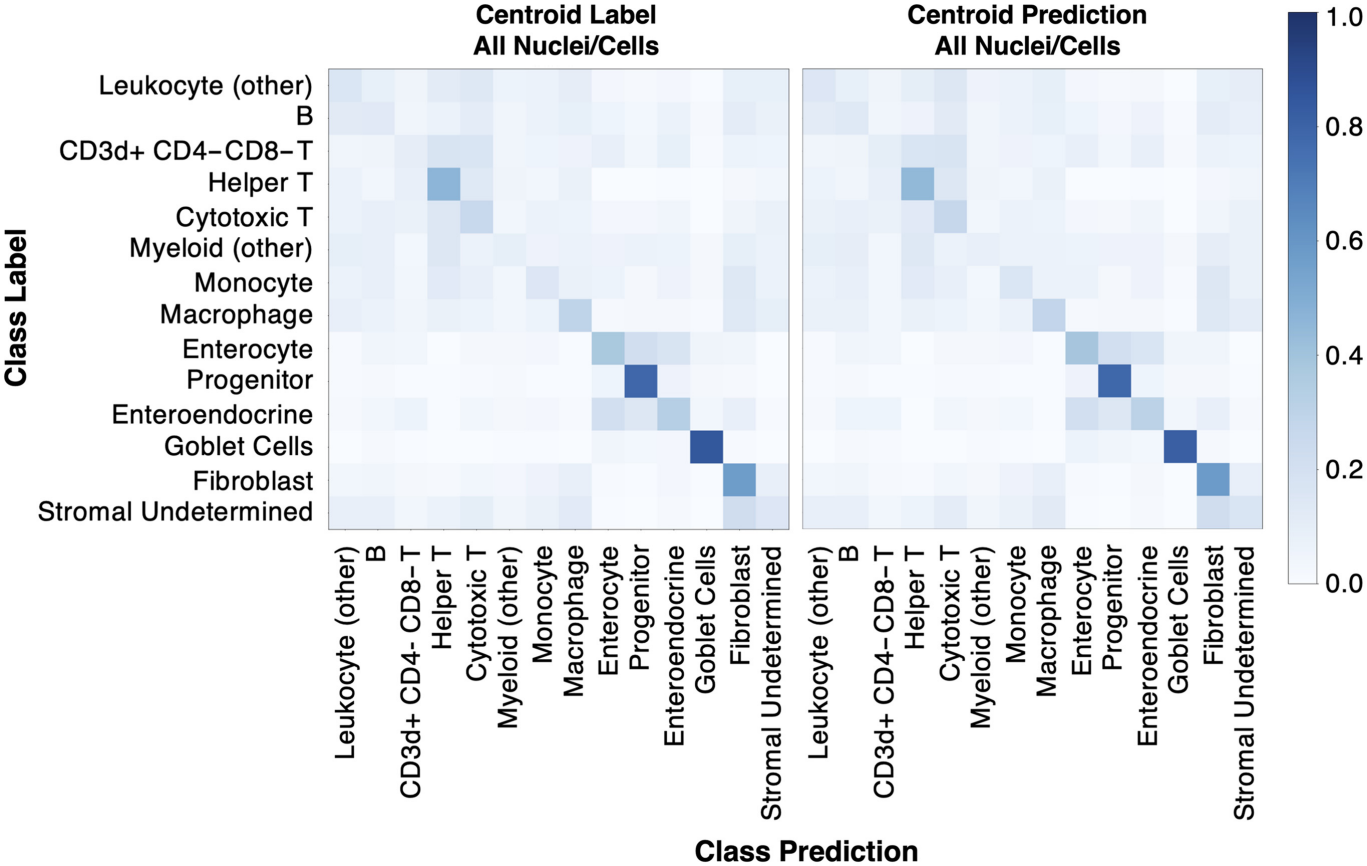
Although not all classes were learned from virtual H&E (shown for fivefold cross-validation), learning behavior can be seen for helper T, macrophage, enterocyte, progenitor, enteroendocrine, and fibroblast nuclei, as well as goblet cells. The classification model’s ability to learn MxIF label information (derived from 17 stain channels) on virtual H&E (three RGB channels) implies that there is a signal present in our virtual H&E to learn some of our fine-grained classification subtypes. From left to right, we show classification accuracy is stable when using label centroids and predicted centroids.

On virtual H&E, the classification accuracy of the ResNet showed some learning behavior for a subset of classes (Fig. 6). These classes were helper T, macrophage, enterocyte, epithelial progenitor, enteroendocrine, and fibroblast nuclei, as well as goblet cells. We show that this classification was stable when the classification was performed using the ground truth centroids, as well as when the centroids were predicted with the Hover-Nets.

**Fig. 7:**
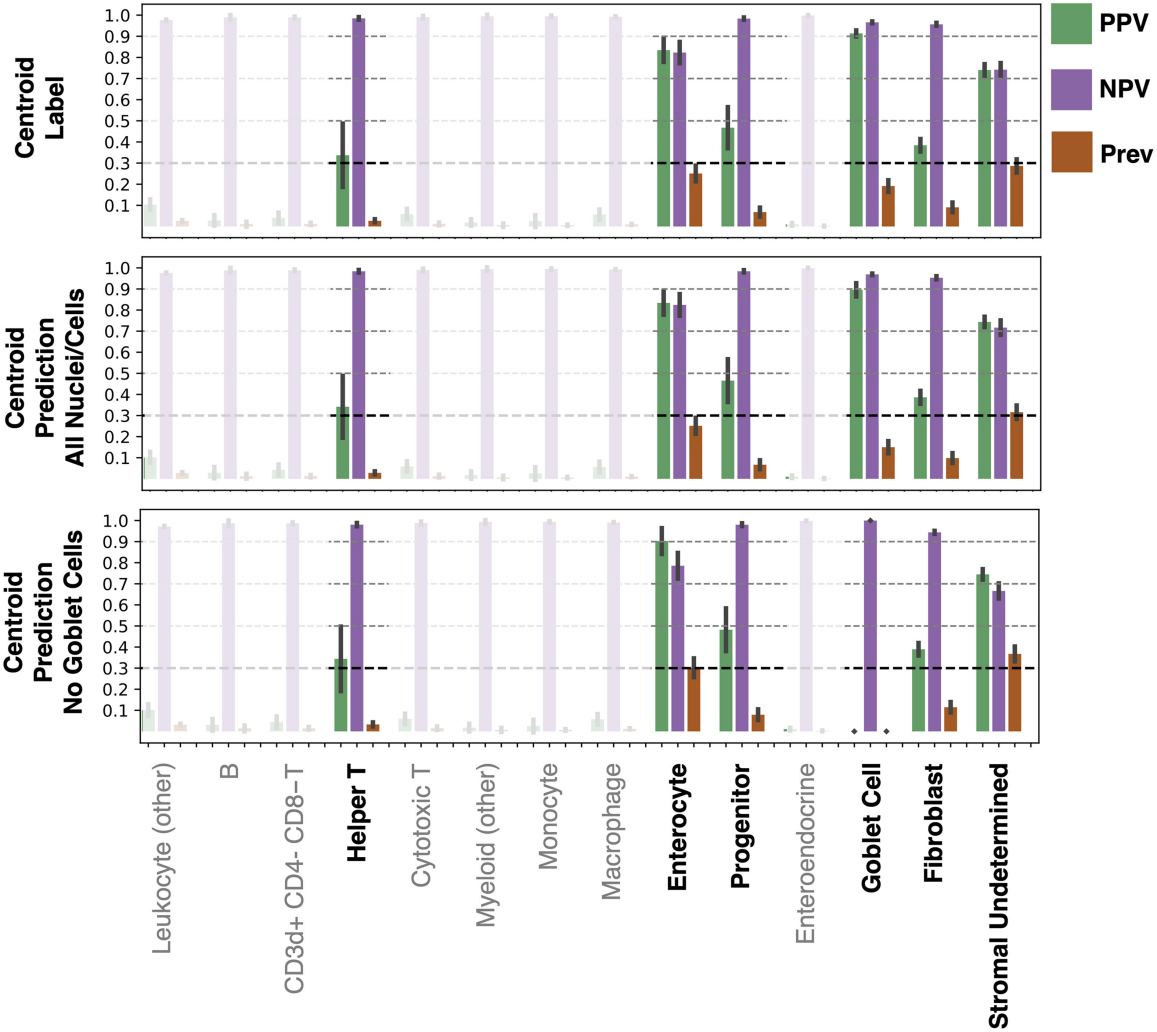
Virtual H&E metrics of PPV, NPV, and prevalence are shown highlighting which classes can be reasonably identified. These metrics give more insight into the usefulness of model predictions than the accuracy shown in a confusion matrix. Helper T, enterocyte, epithelial progenitor, fibroblast, and stromal (undetermined) nuclei, as well as goblet cells, show reasonable learning. In addition, performance is relatively stable when comparing classification using ground truth centroids, predicted centroids (all nuclei/cells), or predicted centroids (all except goblet).

**Fig. 8:**
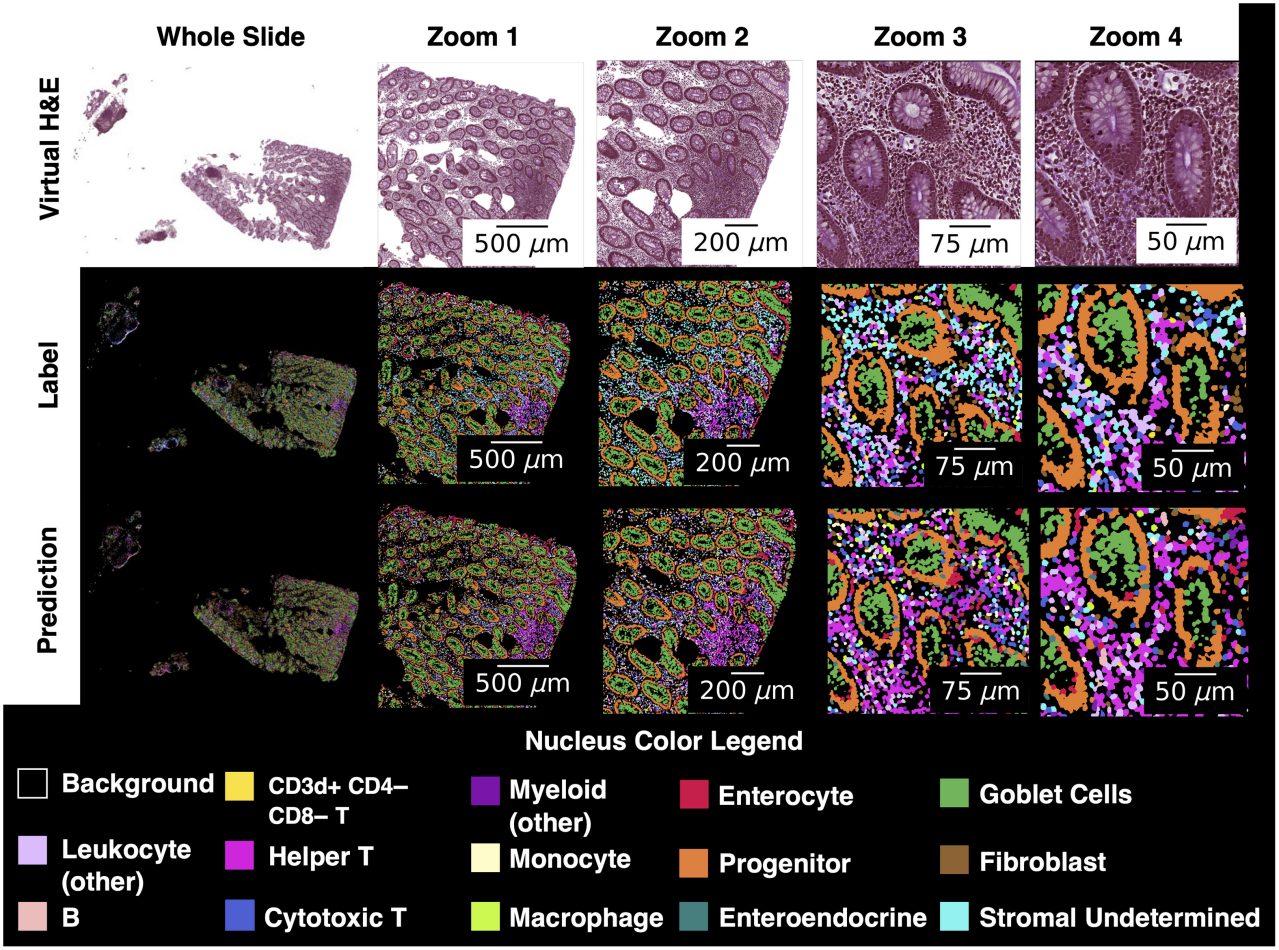
Classification is visually consistent at multiple zoomed scales for enterocyte and progenitor nuclei, as well as goblet cells. Stromal (undetermined) nuclei are often incorrectly classified as a variety of immune cells. Nuclei in an area with immune activity are often correctly identified as helper T cells, although the predictions do include a non-trivial number of false positives for helper T. Crypts, visible here as oval structures at zoomed scales mostly outlined by collections of progenitor (orange) nuclei, are expected to be comprised of epithelial cells. However, progenitors around the crypts in the label set are not surprising because 96% of the progenitors in this dataset are positive for stains that identify epithelium.

Looking in more detail at classification performance on virtual H&E, we computed the positive predictive value (PPV), negative predictive value (NPV), and prevalence (Fig. 7). When prevalence is low, we expect PPV to be low and NPV to be high. Likewise, when prevalence is high, we expect PPV to be high and NPV to be low. A cutoff for reliable classification could be selected based on PPV (Fig. 7 at 0.3). The cutoff of PPV at 0.3 was selected based on visual inspection of Fig. 7. The classes above this threshold are helper T, enterocyte, epithelial progenitor, fibroblast, and stromal (undetermined) nuclei, as well as goblet cells. When considering prevalence, we note that PPV is high for helper T and epithelial progenitor nuclei, and NPV is high for goblet cells and enterocyte nuclei. These results are relatively stable when using the ground truth centroids or predicted centroids. Qualitative results of nucleus/cell classification are reasonable and detailed in Fig. 8.

### 3.3 Results for Real H&E Nucleus Classification

We evaluated our virtual-trained models on real H&E, for five of the six classes that showed learning behavior on PPV and NPV (helper T, enterocyte, epithelial progenitor, fibroblast, and stromal undetermined). The sixth class that was not transferred was goblet cells. In our virtual H&E, we identified goblet cells by the goblet, not the nucleus. Because there were no appropriate corresponding labels in the real H&E data (only nuclei were labeled), we excluded goblet cells from this evaluation. We matched our selected virtual H&E classes to the closest matching and available parent classes on the real H&E. The matching scheme between the virtual H&E nucleus classes to real H&E nucleus classes was as follows: helper T to lymphocyte, enterocyte to epithelial, epithelial progenitor to epithelial, fibroblast to connective, and stromal (undetermined) to connective. Because we matched to parent classes, we could not compute PPV and NPV directly and so instead computed bounds on PPV and NPV.

**Fig. 9:**
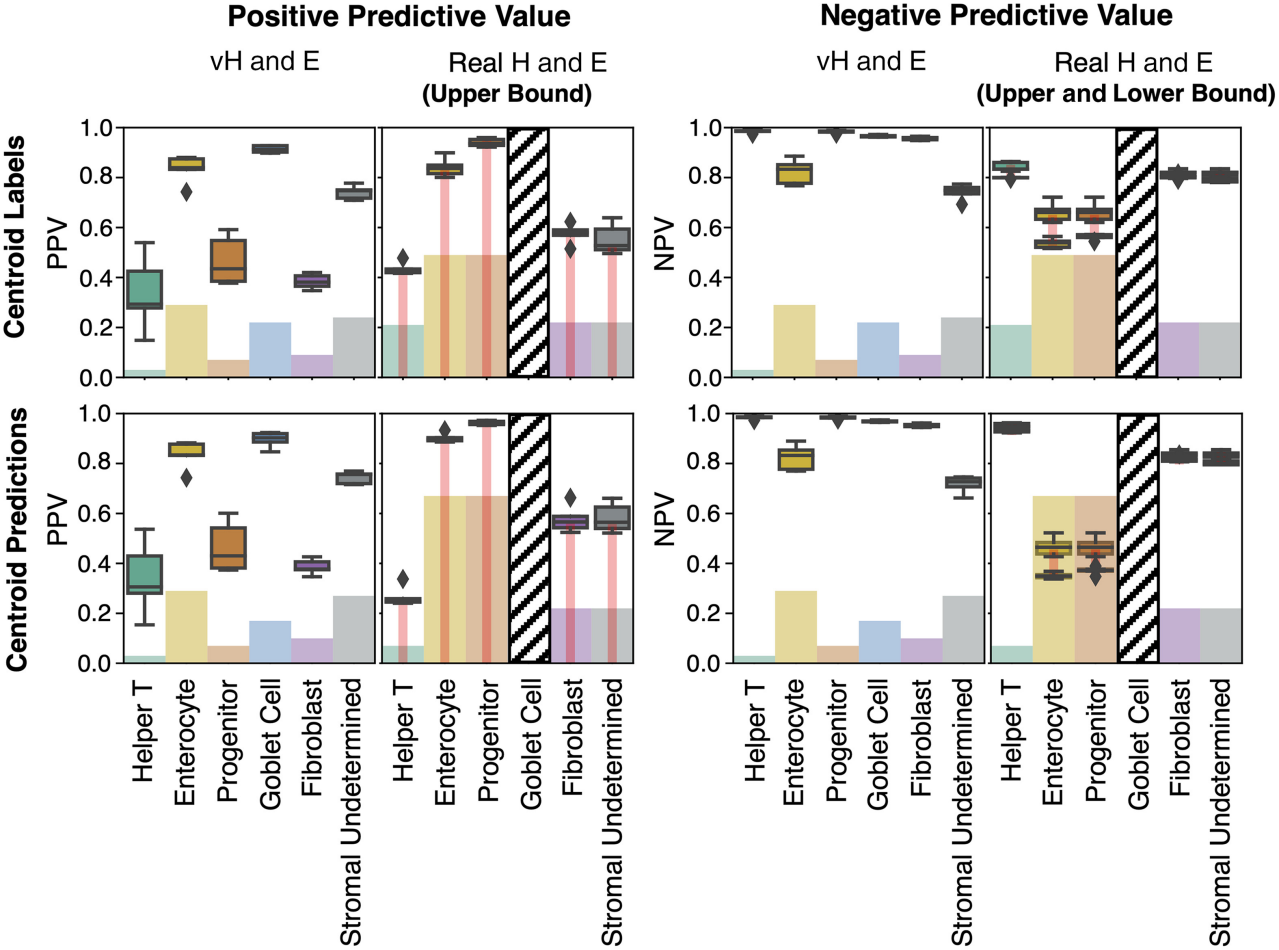
Nucleus classification learned on virtual H&E transfers reasonably well to the real H&E external testing data, despite it being diverse and coming from multiple source datasets. Boxplots for positive predictive value and negative predictive value are shown for both virtual H&E and all source datasets from the real H&E on a subset of cell types. The bar plots denote class prevalence for virtual H&E and the closest matching class prevalence on the real H&E. A thin red connecting bar is used on real H&E to denote that the metrics available are upper bounds on PPV and upper and lower bounds on NPV. Because of the lack of real H&E labels at the granularity of our cells of interest, our NPV and PPV are bounded metrics based on the available parent class labels—this means that we can only compute an upper bound for PPV, which is optimistic, and an upper and lower bound for NPV, which allows for an estimate of NPV. Goblet cell performance is not shown for real H&E because the goblets were not labeled in the real H&E testing data, and so, there was no appropriate available matching label.

**Fig. 10:**
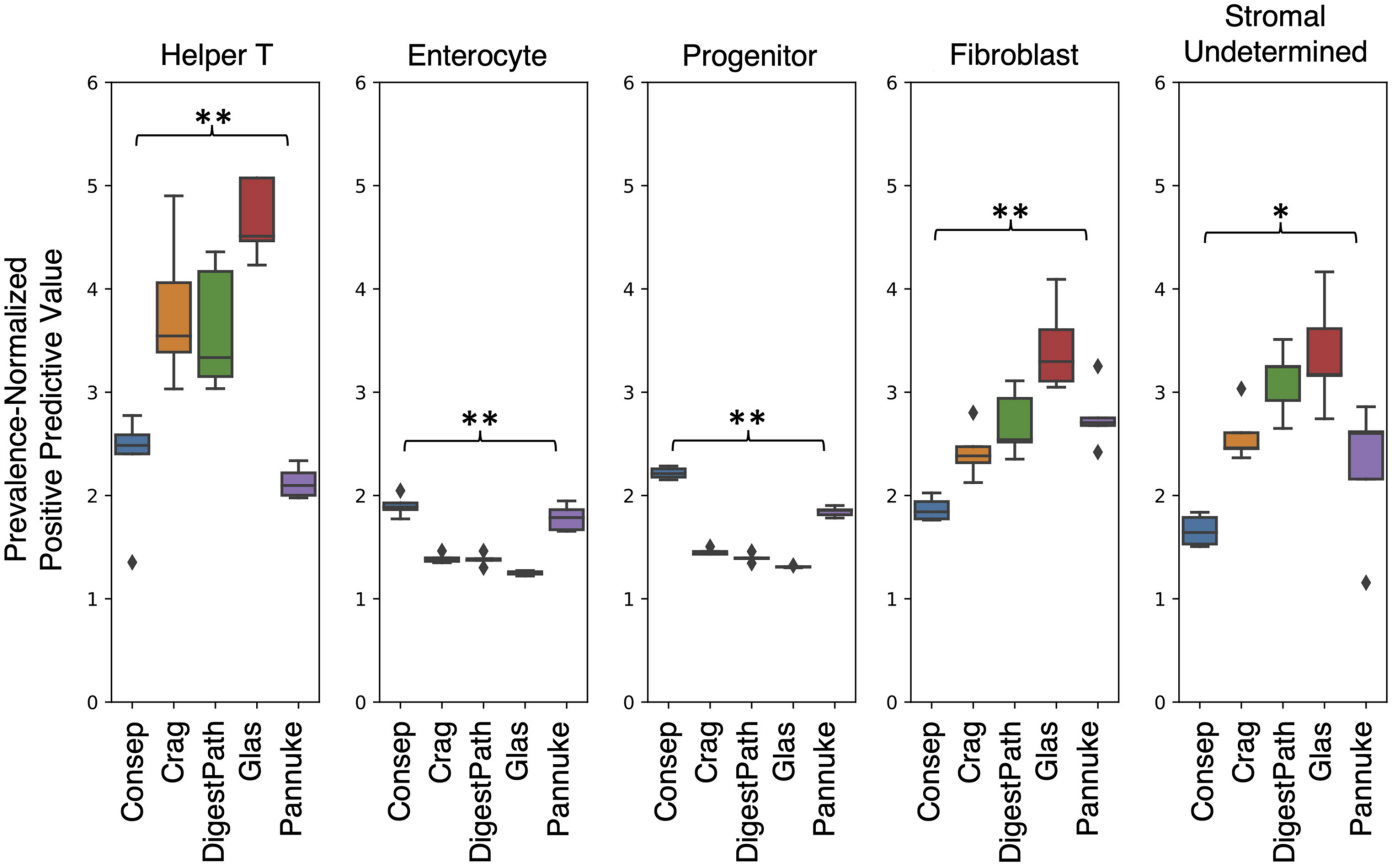
To assess the stability of our virtual-trained cell identification and classification pipeline on real H&E, we evaluated our approach across the source datasets within the real H&E dataset (Consep, Crag, DigestPath, Glas, Pannuke). Because the real H&E does not have labels to the granularity of our shown subclasses (helper T, enterocyte, epithelial progenitor, fibroblast, stromal undetermined), the PPV that we computed is an upper bound based on the matching available parent class labels. To compare the upper bound PPV across datasets for each cell type, we normalized by class prevalence (upper bound PPV divided by prevalence), which varies across source datasets. For each cell type shown, we computed the Friedman test to assess for differences across datasets. Values of p<0.05 are denoted with * and p<0.001 with **. Statistical significance for helper T, enterocyte, epithelial progenitor, fibroblast, and stromal (undetermined) nuclei backs up what is visually evidenced by the boxplots—performance on the PPV upper bound varies across datasets.

Some of the information for some of the classes learned on virtual H&E can transfer to an external testing set of real H&E (Fig. 9). Looking in more detail, we compared performance on different datasets/sites of real H&E by using prevalence-normalized PPV (PPV divided by prevalence). We found that prevalence-normalized PPV showed instability across datasets/sites, despite the use of style transfer to address differences in staining (Fig. 10).

## 4 Discussion and Conclusion

In this paper, we trained a nucleus/cell subclassification model for H&E by leveraging inter-modality learning to train models on virtual H&E with MxIF label information for 14 classes. Identification and classification of nuclei and cells were reasonably learned on virtual H&E for these classes: helper T, epithelial progenitor, enterocyte, fibroblast, and stromal (undetermined) nuclei, as well as goblet cells. Validation was performed on real H&E for helper T, epithelial progenitor, enterocyte, fibroblast, and stromal (undetermined) nuclei.

Although it is feasible to create a large number of labels for nucleus/cell subtypes from MxIF, many of these labels are not easily learned on paired H&E-like data. This is not surprising as specialized stains are commonly used to isolate many of the nuclei/cells we attempted to learn to identify, such as helper T cells. However, for helper T, epithelial progenitor, enterocyte, fibroblast, stromal (undetermined) nuclei, and goblet cells, there is some learnable information in our virtual H&E.

Due to the lack of previous works performing multi-class nucleus classification on H&E using MxIF stain label information, it is difficult to compare the performance of our model to the literature. In the similar work from Han et al.,^22^ four types of nuclei (i.e., ER+, PR+, HER2+, and, Ki67+) were identified from real H&E information with AUCs≥0.75 using MxIF stain label information. These markers were used to assess breast cancer samples and were not present in our marker panel. In addition, their metric was for binary classification rather than multi-class classification so a direct quantitative comparison is not reasonable.

In Fig. 10, we observed intra-class variability on the prevalence-normalized PPV upper bound across datasets/sites in the real H&E testing data. Although we used a CycleGAN for style transfer to correct for H&E stain variation across sites, perhaps more sophisticated harmonization approaches will be needed in the future to account for inter-site variability. In addition, other causes of site instability may include type of disease, disease severity, and cell type prevalence.

This work is limited by a lack of labels on the real H&E testing data at the same level of cell type granularity as our virtual H&E labels. The lack of real H&E on the same tissue cuts as the MxIF was due to the specific clinical protocol that was followed during the dataset creation. Real H&E was available for the MxIF samples on close-cuts (adjacent tissue slices from the same tissue block); however, due to systematic tissue and nucleus misalignment between close-cuts, we opted for the virtual H&E approach in this paper. A lack of real H&E corresponding to the MxIF, where we had 14 class labels, meant that we needed to evaluate our nucleus subclassification approach on an outside real H&E dataset (CoNIC), which did not have the same fine-grained subclassification labels. Due to the lack of the same depth of subclassification labels in the CoNIC dataset, we were only able to compute bounds on metrics rather than the metrics themselves. A more convincing approach for learning MxIF label information on H&E stains would require having H&E staining and MxIF on the same histological samples (same cut), which would eliminate the need for virtual H&E.

For the classification of cells/nuclei into 14 categories on virtual H&E, we did not directly compare with a baseline model trained on ground truth H&E because we did not have H&E staining on the same tissue cuts from MxIF. H&E being stained on the close-cuts meant that there was a nucleus misalignment with the MxIF-derived labels. In addition, for the classification of nuclei into six classes on the CoNIC dataset, we did not directly compare with a baseline model trained on ground truth H&E because we did not have labels that mapped cleanly into half of the CoNIC classes (plasma, eosinophil, and neutrophil). Moreover, we could not reasonably pool the 14 labels together due to a lack of learning in several of the classes (Figs. 6 and 7). In this paper, we did not aim to train a better H&E model than a CoNIC-trained model, rather we aimed to characterize the extent to which we could identify these 14 subcategories of cells/nuclei available from MxIF stains.

Although our virtual H&E appears structurally realistic, the stain color is a fairly monochromatic red/purple. The creation of the virtual H&E was performed using real H&E close-cut data from the same site as the MxIF; however, the real H&E was stained in different batches that had systematic stain variations across batches. The monochromatic red/purple stain coloring in the virtual H&E may be due to an average interpretation of the H&E stain differences across batches in the CycleGAN approach.

**Fig. 11:**
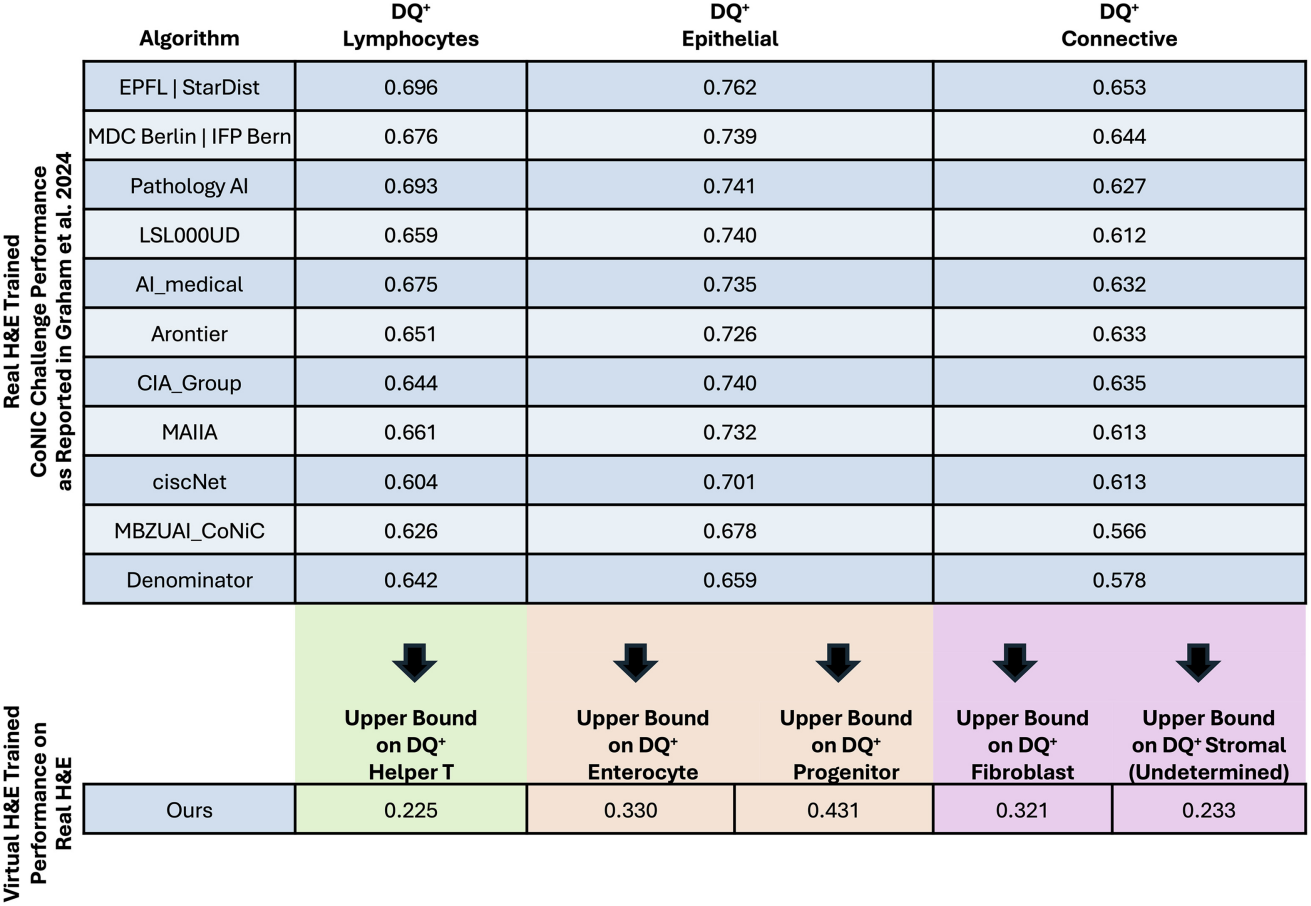
To provide context for our model’s classification performance on real H&E images after being trained solely on virtual H&E, we compared it to detection quality (${\mathrm{DQ}}^{+}$) results from the literature. The top portion of the figure contains results from Graham et al.’s wrap-up publication from the CoNIC Challenge (trained on real H&E and evaluated on real H&E).^38^ The bottom portion of the figure denotes the performance of our approach (trained on virtual H&E and evaluated on real H&E). Because we did not have direct matching labels between our fine-grained virtual H&E labels and the coarser real H&E labels in the CoNIC dataset, we computed an upper bound on ${\mathrm{DQ}}^{+}$ for corresponding classes. The mapping between parent classes (top) and subclasses (bottom) is denoted via color blocks and downward arrows. It is important to note that the results in the upper portion of the figure were computed on different data than the metrics on the bottom portion of the figure and so must be leniently compared, rather than directly compared. Despite limitations for direct comparison, there was a large gap in performance between the real H&E-trained models on parent classes and our virtual H&E-trained model on subclasses. Although we expected the virtual trained model to have greatly reduced performance due to domain shift and weak mapping of labels across the virtual H&E and real H&E datasets, our model is still progressing on the H&E nucleus subtyping frontier.

In Fig. 11, we highlight the limitations of our virtual approach with a lenient comparison to the real H&E literature using detection quality (${\mathrm{DQ}}^{+}$) to assess nucleus classification. The performance of our model, trained on virtual H&E, was substantially reduced compared with submissions to the CoNIC Challenge that were trained on real H&E.^38^ Several factors contributed to the reduction in performance, including a domain shift from virtual to real H&E and the challenge of mapping fine-grained subclass labels from the virtual data to the coarser parent labels on real H&E. In addition, as the real H&E models from the literature were evaluated on different data (our virtual H&E models were trained on in-house virtual data and evaluated on the CoNIC Challenge training data), a direct comparison is not possible, which is why we have reserved Fig. 11 for the discussion section. Despite the more challenging setup for our virtually trained model, the performance gap was not surprising. We expect real H&E models to work better on real H&E than models trained on virtual data. Although our model showed limitations in classification (${\mathrm{DQ}}^{+}$) performance compared with real H&E models for the coarse nucleus parent class labels, our model still represents a meaningful step forward in subtyping nuclei on H&E.

Classification of nuclei and cells on H&E is promising for helper T, epithelial progenitor, enterocyte, fibroblast, stromal (undetermined) nuclei, and goblet cells. The ability to discern nucleus/cell subtypes based on shape and H&E staining is an exciting prospect in computational pathology. We have released code related to this paper at this online repository: github.com/MASILab/nucleus_and_cell_classification_on_he.

## Data Availability

The H&E data from the CoNIC Challenge are publicly available here: https://conic-challenge.grand-challenge.org/. Code from this work is available on GitHub here: https://github.com/MASILab/nucleus_and_cell_classification_on_he or alternatively on Zenodo here https://doi.org/10.5281/zenodo.13338626.
